# Application of Micropore Device for Accurate, Easy, and Rapid Discrimination of *Saccharomyces pastorianus* from *Dekkera* spp.

**DOI:** 10.3390/bios11080272

**Published:** 2021-08-12

**Authors:** Kazumichi Yokota, Asae Takeo, Hiroko Abe, Yuji Kurokawa, Muneaki Hashimoto, Kazuaki Kajimoto, Masato Tanaka, Sanae Murayama, Yoshihiro Nakajima, Masateru Taniguchi, Masatoshi Kataoka

**Affiliations:** 1 Health and Medical Research Institute, National Institute of Advanced Industrial Science and Technology (AIST), 2217-14 Hayashi-cho, Takamatsu, Kagawa 761-0395, Japan; kazumichi-yokota@aist.go.jp (K.Y.); abe-abe@aist.go.jp (H.A.); muneaki-hashimoto@aist.go.jp (M.H.); k-kajimoto@aist.go.jp (K.K.); mst-tanaka@aist.go.jp (M.T.); y-nakajima@aist.go.jp (Y.N.); 2 Institute for Future Beverages, Research & Development Division, Kirin Holdings Company, Limited. 1-17-1, Namamugi, Tsurumi-ku, Yokohama, Kanagawa 230-8628, Japan; Asae_Takeo@kirin.co.jp (A.T.); y-kurokawa@kirin.co.jp (Y.K.); 3 The Institute of Scientific and Industrial Research, Osaka University, 8-1 Mihogaoka, Ibaraki, Osaka 567-0047, Japan; murayama@sanken.osaka-u.ac.jp (S.M.); taniguti@sanken.osaka-u.ac.jp (M.T.)

**Keywords:** resistive pulse method, yeast, discrimination, brewer, *Saccharomyces pastorianus*, *Dekkera* spp.

## Abstract

Traceability analysis, such as identification and discrimination of yeasts used for fermentation, is important for ensuring manufacturing efficiency and product safety during brewing. However, conventional methods based on morphological and physiological properties have disadvantages such as time consumption and low sensitivity. In this study, the resistive pulse method (RPM) was employed to discriminate between *Saccharomyces pastorianus* and *Dekkera anomala* and *S. pastorianus* and *D. bruxellensis* by measuring the ionic current response of cells flowing through a microsized pore. The height and shape of the pulse signal were used for the simultaneous measurement of the size, shape, and surface charge of individual cells. Accurate discrimination of *S. pastorianus* from *Dekkera* spp. was observed with a recall rate of 96.3 ± 0.8%. Furthermore, budding *S. pastorianus* was quantitatively detected by evaluating the shape of the waveform of the current ionic blockade. We showed a proof-of-concept demonstration of RPM for the detection of contamination of *Dekkera* spp. in *S. pastorianus* and for monitoring the fermentation of *S. pastorianus* through the quantitative detection of budding cells.

## 1. Introduction

The resistive pulse method (RPM) is used for evaluating the transient ionic current blockade associated with the translocation of individual nano- to microsized particles passing through an appropriate diameter pore. Moreover, it is applicable in proving small objects by using pulse-like electrical signals. In addition, because the measured ionic current blockade signals possess information regarding the properties of these particles such as size [1], shape [2,3,4], surface charge [1,5], and deformability [6,7], these objects can be discriminated at a single-particle resolution. Further, single bioparticles of various sizes ranging from blood cells to polynucleotides can be discriminated against without implementing immunostaining [2,8,9]. RPM with microsized pores (micropore devices), which is similar to the operating principles of a Coulter counter, is widely used to measure the number of blood cells in hematological diagnosis.

In brewing, traceability analysis such as identification and/or discrimination of microorganisms used for fermentation is important to ensure manufacturing efficiency and product safety [10,11]. The genus *Saccharomyces* is well-known as the key yeast species in beer fermentation, and *S. pastorianus* is used in bottom-fermented beer [12]. Although the presence of *Brettanomyces* yeasts (teleomorph *Dekkera*) including *Dekkera anomala* and *D. bruxellensis* is encouraged in several types of beer, these yeasts cause contaminations of most beer leading to spoilage [12,13].

Conventional methods used for the detection and/or isolation of yeast contamination are based mainly on morphological and physiological properties [14]. Several types of selective media are commonly used to identify and discriminate unknown yeasts. However, these cultivation methods require several days to obtain results. Although gene analysis methods based on PCR are also employed to identify and/or discriminate brewing yeasts [14,15], some disadvantages remain. For example, several hours are required to acquire results and complex operations. Therefore, detection methods for unwanted yeast contamination in brewing with rapid and easy operation, such as in-line inspection, are necessary.

In this study, we developed a micropore device for the accurate and rapid discrimination of *S. pastorianus* from *Dekkera* spp. The budding state of *S. pastorianus* can also be detected at the single-cell level by analyzing the shape of the current blockade signals.

## 2. Materials and Methods

### 2.1. Yeast Strains and Preparation

*S. pastorianus* W34/70, *D. anomala* DSMZ 70727, and *D. bruxellensis* NBRC 0677 were used in this study. These yeasts were cultivated in YPAD agar plates [16] (Thermo Fisher Scientific, Inc., Waltham, MA, USA) for at least 2 days. To prepare the cell sample solution for ionic current measurements, appropriate amounts of each yeast were collected from a single colony and suspended in 50 mM 2-(N-morpholino) ethanesulfonic acid (MES) buffer to a concentration of 1 × 10^6^ cells/mL.

### 2.2. Cell Size Measurement Using Light Microscope

**Figure 1:**
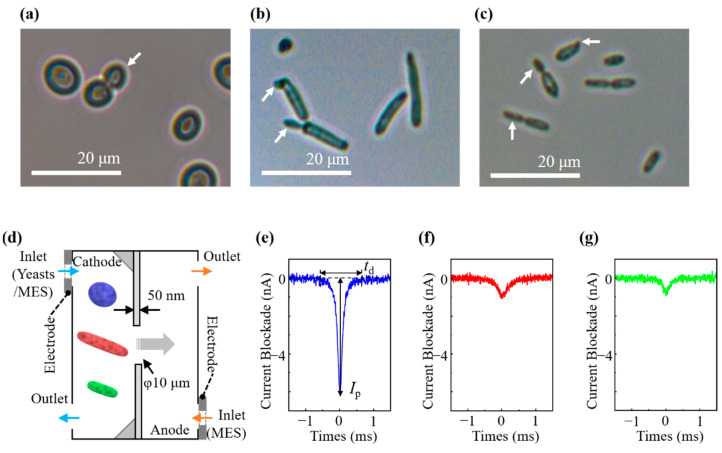
Light microscopic images of (**a**) *S. pastorianus*, (**b**) *D. anomala*, and (**c**) *D. bruxellensis*. Budding is observed in each. Arrows indicate daughter cells. (**d**) Picture of micropore device with a diameter of 10 μm. A typical waveform of current ionic blockade by RPM was observed for (**e**) *S. pastorianus*, (**f**) *D. anomala*, and (**g**) *D. bruxellensis*.

One hundred cells in each cell suspension in 50 mM MES buffer were selected using a light microscope (DIML II, Leica Camera AG, Wetzlar, Germany) with a 40× objective lens to determine each yeast size. Approximately 20 μL of each cell sample was seeded into a 96-well plate (Nunc MicroWell 96-Well Microplates, Thermo Fisher, Inc.) to settle at the bottom of the plate surface, and the diameter of each cell was measured immediately [Figure 1a–c]. Cell sizes were expressed as mean ± standard deviation (SD).

### 2.3. Micropore Device and Ionic Current Measurement

For the ionic current measurement of cells, we employed a micropore device with a diameter of 10 μm and thickness of 50 nm, as shown in Figure 1d (M-NK-1000-A106-001-Pm, Aipore, Inc., Tokyo, Japan). Then, 10 μL of cell sample solution was injected into the cathode-side microchamber through the inlet, and 10 μL of 50 mM MES buffer was injected into the anode-side microchamber through the inlet. For cell mixture measurement, two of three types of yeast, 5 μL each and 10 μL in total, were injected into the cathode-side microchamber.

A current amplifier (ACDC3000, AXIS NET, Inc., Osaka, Japan) was employed for ionic current measurement in the current range of 2.5 μA using the LabVIEW (LabVIEW 2017, National Instruments, Austin, TX, USA) program. The time trace of the ionic current was evaluated via the pore and was recorded at a sampling rate of 1 MHz using a voltage input module (NI-9223, National Instruments), which corresponds to a 1-μs resolution time. Typical waveforms of the ionic current blockade for *S. pastorianus*, *D. anomala*, and *D. bruxellensis* are shown in Figure 1e–g. The duration of the blockade signal was >0.2 ms, and the employed sampling rate was sufficient to analyze the signal of the current blockade. For each experiment, 400 current blockade signals could be obtained in 6 min.

### 2.4. Resistive Pulse Analysis and Cell Discrimination

We measured resistive pulse signals that appeared on the time trace of the ionic current by monitoring the current displacement, which was larger than the threshold by three times the SD. Further, we averaged the data for the nearest neighboring points to reduce the current noise in the pulse measurement process, as reported by Smeets et al. [17]. In addition, we extracted the waveforms of the pulses of the original 1-MHz data at the time point of pulse detection and evaluated the peak values of the current blockade (*I*_p_) and the duration of the current blockades (*t*_d_) on the extracted waveform [Figure 1e–g]. Four hundred pulse signals were analyzed. These data were processed using LabVIEW.

### 2.5. Discrimination of Decision Boundary and Discrimination Error

Compared with the linear separation boundary [18], a quadratic discrimination analysis (QDA) with a nonlinear separation boundary can discriminate more accurately between different classes. QDA is a probabilistic parametric classification technique that separates the class region by quadratic boundaries assuming that each class has a multivariate normal distribution with the dispersion being different per class. The decision boundary (DB) by QDA for cell discrimination is defined by a contour line/curve providing an equal probability of the *I*_p_, *t*_d_, and *I*_p_-*t*_d_ distributions for each cell [19,20]. The probability is expressed as follows:

$$P\left(\log_{10}y\right)=\frac{1}{\sqrt{2\pi \left|\Sigma_{k}\right|}}\exp\left(-\frac{1}{2}{\left(\log_{10}y-\langle \log_{10}{y\rangle }_{k}\right)}^{T}\Sigma_{k}^{-1}\left(\log_{10}y-\langle \log_{10}{y\rangle }_{k}\right)\right).$$

where Σ*_k_* and $\langle \log_{10}{y\rangle }_{k}$ are the variance-covariance matrix and the mean value of observations $\log_{10}y$ for the *k*th dimension (*k* = $\log_{10}I_{p}$ or $\log_{10}t_{d}$), respectively.

### 2.6. Zeta Potential Measurement

The zeta potential was measured using a zeta potential analyzer (ELSZ-2000Z Otsuka Electronics Co., Ltd., Osaka, Japan). A glass flow cell for measurements was filled with 1.0 mL of cell sample at a concentration of 1 × 10^8^ cells/mL in 50 mM MES buffer. While applying an electric field of ~16 V/cm on average, the electrical mobility was evaluated from the Doppler shift of the scattering light of the laser, and the zeta potential *ζ* was obtained by fitting the electrophoretic velocity of cells flow inside the measurement glass cell based on the Smoluchowski equation [21]. The measured zeta potential is represented by the mean ± SD (*n* = 6).

### 2.7. Multiphysics Simulations of Ionic Current Waveform for a Budding Yeast

To elucidate the characteristic ionic current waveforms originating from the morphological features of budding yeasts passing through a pore, numerical simulations based on finite element methods were conducted [2,4]. The geometric structures were modeled in a cylindrical coordinate system (*r*, *θ*, and *z* as the radial, azimuthal, and axial coordinates, respectively), as shown in Figure 4a. The total model size was 50 μm in radius (*R* = 50 μm) and 100 μm in *z*-height. A membrane with a thickness of 50 nm formed a 5-μm radius pore [white arrow in Figure 4a] and was placed in the middle of the model (*z* = 0 μm).

A yeast model [black arrow in Figure 4a] of a given size (parameterized by *L*_x_) was positioned along the *z*-axis. The multiphysics simulation was conducted by simultaneously solving Equation (1) the continuity equation at a steady-state (∇·j=0, where *j* is the current density) for the applied electric potential of *V*_c_, Equation (2) the Poisson–Boltzmann equation for the electrostatic potential of *V*_s_, Equation (3) the Nernst–Planck equation for the i ion concentration of *c*_i_, and Equation (4) the incompressible Navier–Stokes equation for the hydrodynamic pressure and the flow field of *p* and *U*:

(1) $$\nabla \cdot j=\nabla \cdot \left[-\left(\sigma_{w}+F\underset{i}{\sum }z_{i}^{2}u_{i}c_{i}\right)\nabla V_{c}\right]=0$$

(2) $$\begin{array}{c} \nabla^{2}V_{s}=-\frac{\rho }{\varepsilon_{w}}=-\frac{1}{\varepsilon_{w}}F\underset{i}{\sum }z_{i}c_{i}\exp(-z_{i}eV_{s}/k_{B}T) \end{array}$$

(3) $$\nabla \cdot \left(-D_{i}\nabla c_{i}-z_{i}u_{i}Fc_{i}\nabla V_{s}\right)+U\cdot \nabla c_{i}=0$$

(4) $$-\nabla p+\eta \nabla^{2}U-\rho \nabla V_{c}=0$$

Here, *σ*_w_, *F*, *z*_i_, and *u*_i_ are the electrical conductivity of water, the Faraday constant, charge number, and electrical mobility of ion i, respectively. We used *z*_i_ = 1 and *u*_i_ = 3.69 × 10^−7^ m^2^/V∙s for i = H^+^, and *z*_i_ = −1 and *u*_i_ = 2.87 × 10^−8^ m^2^/V∙s for i = MES^−^. These *u*_i_ values were evaluated from the diffusion coefficient of *D*_i_ [22,23] based on the Einstein relation *u*_i_ = *eD*_i_/*k*_B_*T*, where *e*, *k*_B_, and *T* are the elementary charge, Boltzmann constant, and temperature, respectively. *ρ*, *ε*_w_, and *η* are the net ionic charge density, permittivity, and dynamic viscosity of water, respectively.

The boundary conditions for Equation (1) [*V*_c_ at *z* = 50 μm and −50 μm] were 0.1 V and 0 V, respectively. For Equation (2), a surface charge of −0.015 C/m^2^, estimated from the zeta potential by using the Grahame equation [24,25], was imposed on the surfaces of the pore and the yeast model. For Equation (3), the boundaries at *z* = ±50 μm and *r* = 50 μm were assumed to be *c*_i_ = 2.6 mM. This *c*_i_ value was estimated based on the assumption that the total ionic current was 100 nA, which corresponded to the experimentally measured baseline current. The other boundary conditions were similar to those in a previous study [2].

The resulting electric potential (*V*_c_ + *V*_s_) distribution is shown in Figure 4a. The total ionic current *I* was calculated by the surface integral for *j* in the *z*-direction (*j*_z_) at *z* = 50 μm over the *r–θ* surface, expressed as

(5) $$I=\int_{0}^{R}rdr\int_{0}^{2\pi }d\theta j_{z}$$

All numerical simulations were performed with COMSOL Multiphysics 5.0 (COMSOL, Inc., Stockholm, Sweden) using the physical parameters of *σ*_w_ = (18.2 MΩ·cm)^−1^, *F* = 9.649 × 10^4^ C/mol, *e* = 1.602 × 10^−19^ C, *k*_B_ = 1.381 × 10^−23^ J/K, *T* = 293.15 K, *ε*_w_ = 7.097 × 10^−10^ F/m, and *η =* 1.002 × 10^−3^ Ns/m^2^ [23].

## 3. Results and Discussion

### 3.1. Morphological Examination by Light Microscopy

Light microscopic images of cells are shown in Figure 1a–c. *S. pastorianus* cells were round to ovoid shapes with a size of 5.6 ± 0.9 × 6.9 ± 1.2 μm [Figure 1a]. Both *D. anomala* and *D. bruxellensis* were spindle-shaped at 2.9 ± 0.8 × 13.6 ± 5.7 μm and 2.5 ± 0.5 × 7.9 ± 3.2 μm, respectively [Figure 1b,c]. The size and shape of *S. pastorianus* were clearly different from those of *D. anomala* and *D. bruxellensis*. Budding was observed in some cells [Figure 1a–c].

### 3.2. Ionic Current Measurement of Cells and Cell Discrimination

We applied a bias voltage of *V*_b_ = 0.1 V between the cathode-side and the anode-side microchambers, and the baseline had a current level of approximately 100 nA. The electrical conductivity (*σ*) was determined to be 0.104 ± 0.06 S/m. The electric field generated by the bias voltage yields electric forces that drive the cells and translate them through the pores. As illustrated in Figure 1d, the cells were restricted by a pore, causing them to pass turn by turn through the pore, thus yielding a one-to-one correspondence of a pulselike signal between the ionic current blockade and a translocation event of a cell through the pore.

Figure 1e–g depict typical waveforms of the current blockades generated by *S. pastorianus*, *D. anomala*, and *D. bruxellensis*, respectively. The size and deformability of the sensed particles were estimated from the *I*_p_ values generated by the RPM [1,6,7]. The *I*_p_ values of *S. pastorianus*, *D. anomala*, and *D. bruxellensis* were 6.16 ± 3.96 nA, 0.99 ± 0.58 nA, and 0.75 ± 0.46 nA, respectively, which are significantly different. *t*_d_ can be utilized to elucidate the surface charge of the sensed particles [1,5]. The zeta potential was examined to estimate the surface charge of the cells. The evaluated zeta potentials for *S. pastorianus*, *D. anomala*, and *D. bruxellensis* were −28.99 ± 0.50 mV, −16.50 ± 0.64 mV, and −14.42 ± 0.90 mV, respectively. Contrary to the prediction from the results of zeta potential measurement, the *t*_d_ values were 1.31 ± 0.89 ms, 1.07 ± 1.12 ms, and 0.93 ± 0.90 ms with statistical differences between *S. pastorianus*, *D. anomala*, and *D. bruxellensis*, respectively. Since the *I*_p_ values for *Dekkera* spp. are extremely smaller than that of *S. pastorianus*, it is considered that *Dekkera* spp. pass through the pore with a longitudinal direction. The minor axis of *Dekkera* spp. is less than half that of *S. pastorianus*, and the spindle-shaped *Dekkera* spp. are thought to be more mobile than the round shape of *S. pastorianus* in solution, so it may be faster to pass the pore [26]. Therefore, it may be that the *t*_d_ values indicating the residence time in the pore becomes shorter in long *Dekkera* spp. compared with short *S. pastorianus*.

**Figure 2:**
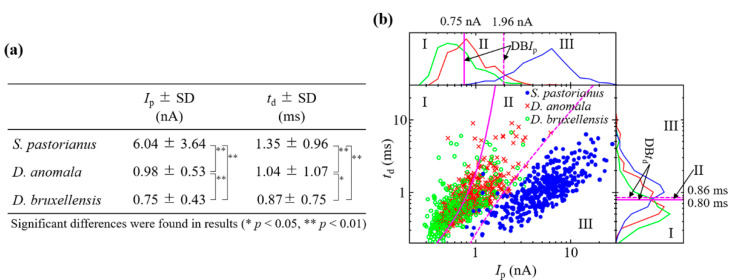
Reproducibility of *I*_p_ and *t*_d_ measurement by RPM for (**a**) *S. pastorianus*, *D. anomala*, and *bruxellensis*. (**b**) Histogram of *I*_p_ on log, *t*_d_ on log, *I*_p_-*t*_d_ on log-log, and DBs for *S. pastorianus*, *D. anomala*, and *D. bruxellensis*.

**Figure 3:**
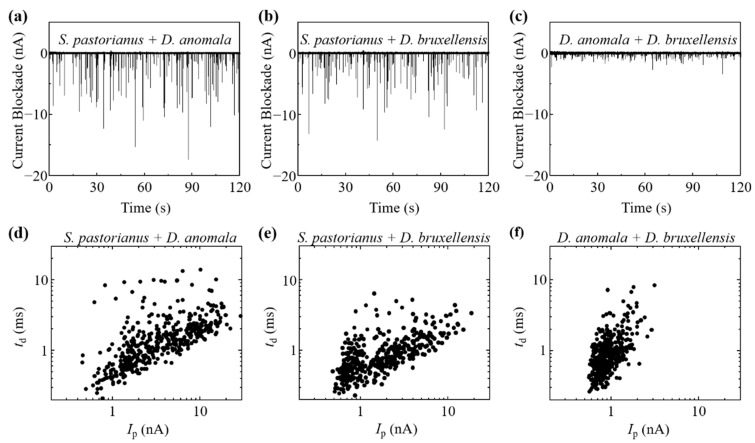
Electrical current record by RPM of each mixture of (**a**) *S. pastorianus* and *D. anomala*, (**b**) *S. pastorianus* and *D. bruxellensis*, and (**c**) *D. anomala* and *D. bruxellensis*. *I*_p_-*t*_d_ scatter plot on log-log graph by RPM of each mixture of (**d**) *S. pastorianus* and *D. anomala*, (**e**) *S. pastorianus* and *D. bruxellensis*, and (**f**) *D. anomala* and *D. bruxellensis*.

The reproducibility of *I*_p_ and *t*_d_ measurements by the RPM for these yeasts was confirmed by three different experiments [27] [Figure 2a]. The histogram of *I*_p_ on log, *t*_d_ on log, and scatter plot of *I*_p_–*t*_d_ on log-log, and the BDs for each cell are shown in Figure 2b. If cells are present in regions I, II, and III on each graph, then they are estimated as *D. bruxellensis*, *D. anomala*, and *S. pastorianus*, respectively. The electrical current records and *I*_p_-*t*_d_ scatter plot by RPM of a mixture of two types of yeast from *S. pastorianus*, *D. anomala*, and *D. bruxellensis* are shown in Figure 3. Large variations in current blockade were observed in each mixture of *S. pastorianus* and *D. anomala* [Figure 3a], and *S. pastorianus* and *D. bruxellensis* [Figure 3b]. Relatively uniform small current blockades could be observed in the mixture of *D. anomala* and *D. bruxellensis* [Figure 3c]. The cell distribution on the *I*_p_-*t*_d_ scatter plot by RPM analysis of cell mixture and single measurement [Figure 2b] is very similar and has high applicability of RPM for accurate discrimination of *S. pastorianus* from *Dekkera* spp. can be expected.

Recall rates of *S. pastorianus, D. anomala,* and *D. bruxellensis* were estimated on log_10_*I*_p_ to be 92.4 ± 1.9%, 54.5 ± 5.3%, and 62.6 ± 3.1%, respectively [Figure 2b and Table 1]. *S. pastorianus* was detected with high sensitivity. On the other hand, recall rates of *S. pastorianus, D. anomala,* and *D. bruxellensis* were estimated to be 56.8 ± 8.9%, 19.5 ± 13.2%, and 66.8 ± 5.4% on log_10_*t*_d_, respectively. Highly sensitive detection of *S. pastorianus* was not observed. By performing discriminant analysis on the log_10_*I*_p_–log_10_*t*_d_ plane, a significant improvement in the recall rate for *S. pastorianus* was 96.3 ± 0.8% compared to log_10_*I*_p_ and log_10_*t*_d_ (McNemar test, *p* < 0.05). The limit of detection was 2.4%, which gave a signal at 3SDs above the backgrounds. The accuracy of *S. pastorianus* identification by PCR is reported to be 94.6% [12]. RPM can be expected to be as accurate as PCR. These results indicate the potential of *I*_p_–*t*_d_ analysis by RPM for the quantitative detection of *S. pastorianus* in solution and the accurate detection of *Dekkera* spp. contamination.

### 3.3. Analysis of Budding S. pastorianus

**Figure 4:**
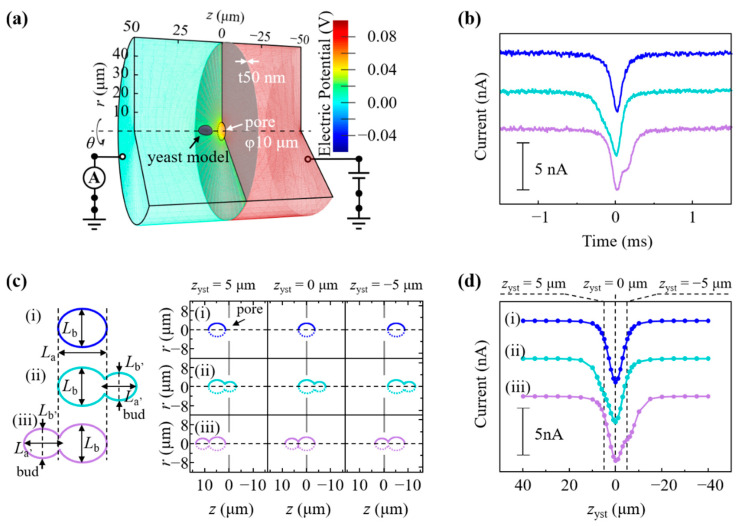
(**a**) Geometric structure modeled in a cylindrical coordinate system. *r*, *θ*, and *z* indicate coordinate, azimuthal angle, and axial coordinate, respectively. The color scale at the right represents the electric potential. (**b**) Measured symmetric waveform (blue), waveform with the shoulder before peak time (cyan), and waveform with a shoulder after peak time (purple). (**c**) Simulated model of passing through the pore of the nonbudding cell (i) and budding cell with daughter in front of (ii) or behind (iii) mother cell. (**d**) Simulated waveforms of the nonbudding cell (i), budding cell with daughter in front of (ii) or behind (iii) mother cell.

In the RPM analysis for *S. pastorianus*, 87.6% of the waveforms [Figure 4b, blue] were symmetrical, and 11.8% had shoulders before the peak time [Figure 4b, cyan] of the waveform and 0.6% after [Figure 4b, purple]. As shown in Figure 1a, budding was observed in some cells. Budding was observed in 11.9% of *S. pastorianus* on microscopic images (data not shown), which is close to the appearance rate of the shoulder-shaped waveform in the RPM.

We simulated whether the shoulder shape of the waveform shows budding by using multiphysics simulations. The size parameter *L*_x_ for the yeast model is a variable that defines the distribution of *V*_c_, *V*_s_, *c*_i_, *p*, and *U*, including *j* and *ρ* in Equations (1)–(4), and affect *I* in Equation (5) [28,29]. In Figure 4c, the blue ellipsoid with *L*_a_ = 6.9 μm on the major axis and *L*_b_ = 5.6 μm on the minor axis represents a nonbudding *S. pastorianus* (i). The budded *S. pastorianus* (cyan and purple) was modeled by merging a bud (daughter cell) to the forward and backward of the mother cell (ii and iii). The sizes of the mother cell (*L*_a_ and *L*_b_) and those of daughter cells (*L*_a’_ = 5.0 μm and *L*_b’_ = 4.1 μm for cyan, and *L*_a’_ = 5.6 μm and *L*_b’_ = 4.5 μm for purple) were assumed from the microscopic images. Figure 4d shows the change in *I* during the yeast *z-*position displacement of *z*_yst_ = 40 to −40 μm. The peak of the current blockade appeared at *z*_yst_ = 0 μm [Figure 4d, i–iii]. The gap of the pore by passing through cells was minimized in the cell model size of *L*_a_ and *L*_b_ at *z*_yst_ = 0 μm [Figure 4c, i–iii]. In contrast to the symmetric shape of (d, i), the shoulder appeared at *z*_yst_ > 0 for (d, ii) and *z*_yst_ < 0 for (d, iii). These corresponded to the passage of the budded *S. pastorianus* model through the pore in the order of daughter–parent (c, ii) or parent-daughter (c, iii), and the appearance of the shoulder was related to the daughter cell size. The simulated waveforms [Figure 4d] were in good agreement with the experimentally measured waveforms [Figure 4b]. Budding *S. pastorianus* can be quantitatively detected by measuring the shouldered waveform of the current ionic blockade. The contributions of the size and orientation of yeasts to the waveform can explain the RPM for the investigated *Dekkera* spp., in which clear shoulders were not seen. *Dekkera* spp. has an elongated spindle shape, and the major axis of *D. anomala* is larger than the pore diameter [Figure 1b,c]. This restricts the orientation of those during passage through the pore in the longitudinal direction, and the cell passes through the pores with its major axis parallel to the z-axis. Since the minor axis of *Dekkera* spp. is apparently smaller than that of *S. pastorianus* [Figure 1a–c], their obtained *I*_p_ values obtained are smaller than those of *S. pastorianus* [Figure 1e–g]. The current resolution to discriminate the budding cells in *Dekkera* spp. may be insufficient by the RPM analysis employed in this study. A smaller-sized pore is necessary to improve the resolution for analyzing the budding of *Dekkera* spp. These results indicate the potential application of RPM for the monitoring of growth viability and fermentation of yeasts by the quantitative detection of budding cells [30,31,32].

## 4. Conclusions

In this study, we demonstrated the potential of RPM analysis using a micropore device with a 10-μm diameter pore for accurate discrimination of *S. pastorianus* from *Dekkera* spp. by measuring multiple parameters such as cell size, and the quantitative detection of budding *S. pastorianus* by evaluating the shape of the waveform of the current ionic blockade. We demonstrated the proof-of-concept of RPM for the detection of *Dekkera* spp. contamination in *S. pastorianus* and for monitoring the fermentation of *S. pastorianus* through the quantitative detection of budding cells.

## Figures and Tables

**Table 1:** Discrimination of investigated yeasts based on log_10_*I*_p_ acquired by RPM.

		Predicted Classification			
		*S. pastorianus*	*D. anomala*	*D. bruxellensis*	Total
Actual classification	*S. pastorianus*	376 372 361	22 28 39	2 0 0	400 400 400
	Recall	92.4 ± 1.9%			
	*D. anomala*	19 25 23	242 209 203	139 166 174	400 400 400
	Recall		54.5 ± 5.3%		
	*D. bruxellensis*	10 10 8	138 128 155	252 262 237	400 400 400
	Recall			62.6 ± 3.1%	
Discrimination of investigated yeasts based on log_10_*t*_d_ acquired by RPM.					
		**Predicted Classification**			
		*S. pastorianus*	*D. anomala*	*D. bruxellensis*	Total
Actual classification	*S. pastorianus*	204 268 209	88 15 32	108 117 159	400 400 400
	Recall	56.8 ± 8.9%			
	*D. anomala*	100 178 113	106 17 111	194 205 176	400 400 400
	Recall		19.5 ± 13.2%		
	*D. bruxellensis*	73 97 101	80 13 34	247 290 265	400 400 400
	Recall			66.8 ± 5.4%	
Discrimination of investigated yeasts based on log_10_*I*_p_–log_10_*t*_d_ acquired by RPM.					
		**Predicted Classification**			
		*S. pastorianus*	*D. anomala*	*D. bruxellensis*	Total
Actual classification	*S. pastorianus*	383 389 384	15 11 14	2 0 2	400 400 400
	Recall	96.3 ± 0.8%			
	*D. anomala*	11 12 5	252 247 243	137 141 152	400 400 400
	Recall		61.8 ± 1.1%		
	*D. bruxellensis*	3 4 1	139 139 62	258 257 337	400 400 400
	Recall			71.0 ± 11.5%	

## Data Availability

Not applicable.
